# Thrombospondin 2, matrix Gla protein and digital analysis identified distinct fibroblast populations in fibrostenosing Crohn’s disease

**DOI:** 10.1038/s41598-024-64672-7

**Published:** 2024-06-14

**Authors:** Miha Jerala, Tinkara Remic, Nina Hauptman, Pia Homan, Neža Zajšek, Mathieu Petitjean, Li Chen, Nina Zidar

**Affiliations:** 1 Faculty of Medicine, Institute of Pathology, University of Ljubljana, Korytkova 2, 1000 Ljubljana, Slovenia; 2 PharmaNest Inc., Princeton, NJ 08540 USA

**Keywords:** Crohn’s diseases, Fibrostenosis, Myofibroblasts, Thrombospondin 2, Matrix Gla protein, Digital analysis, Inflammatory bowel disease, Diseases, Pathogenesis

## Abstract

Fibrosis is an important complication in inflammatory bowel diseases. Previous studies suggest an important role of matrix Gla protein (MGP) and thrombospondin 2 (THBS2) in fibrosis in various organs. Our aim was to analyse their expression together with regulatory miRNAs in submucosal and subserosal fibroblasts in ulcerative colitis (UC) and Crohn’s disease (CD) using immunohistochemistry and qPCR. Digital pathology was used to compare collagen fibre characteristics of submucosal and subserosal fibrosis. Immunohistochemistry showed expression of MGP, but not THBS2 in submucosa in UC and CD. In the subserosa, there was strong staining for both proteins in CD but not in UC. qPCR showed significant upregulation of *THBS2* and *MGP* genes in CD subserosa compared to the submucosa. Digital pathology analysis revealed higher proportion of larger and thicker fibres that were more tortuous and reticulated in subserosal fibrosis compared to submucosal fibrosis. These results suggest distinct fibroblast populations in fibrostenosing CD, and are further supported by image analysis showing significant differences in the morphology and architecture of collagen fibres in submucosal fibrosis in comparison to subserosal fibrosis. Our study is the first to describe differences in submucosal and subserosal fibroblast populations, contributing to understanding of the pathogenesis of fibrostenosis in CD.

## Introduction

Intestinal fibrosis is a frequent complication with significant morbidity in inflammatory bowel diseases (IBD)^1^. It is especially significant in Crohn`s disease (CD) where it frequently cause intestinal strictures that requires surgical intervention^1,2^. Although fibrosis is less prominent and with less clinical impact in ulcerative colitis (UC), colon stiffening is often seen in longstanding UC while fibrotic strictures are very rare^3,4^. Fibrosis as well as inflammation in CD involves the entire thickness of the bowel wall, most notably with fibrosis in the submucosa and subserosa. In UC, however, fibrosis and inflammation are generally limited to the mucosa and submucosa^5^.

The main effector cell of fibrosis in the bowel and other organs is the activated myofibroblast^6^. There are many postulated origins of activated myofibroblasts with evidence pointing to origins from residential fibroblasts, pericytes, circulating mesenchymal cells and epithelial and endothelial cells via epithelial/endothelial-mesenchymal transition^7^. Among organs where fibrosis has been thoroughly studied, skin is comparable to the intestinal wall as both represent a barrier separating the body from the environment and both consist of different histological layers. In the skin, there are well characterised fibroblast subpopulations with microanatomical stratification by depth^8–11^. They are mainly divided into papillary (more superficial) and reticular (deep) fibroblasts^8–11^. In recent years, some studies have identified distinct fibroblast subpopulations in the bowel wall as well; however, they focused mainly on fibroblast subpopulations in the lamina propria and submucosa^12–14^.

The gastrointestinal tract is frequently exposed to insults that can cause superficial damage such as infections, toxins and immune reactions^15,16^. To maintain bowel functionality, the response to such damage should be without significant scarring. However, damage that extends beyond the muscularis propria threatens the bowel wall integrity and can lead to perforation, which is a life-threatening event. In this case, fibrosis with scar formation is needed and represents a strong protective response^17^. Besides fibrosis, a defining characteristic of CD compared to UC is transmural inflammation, which traverses the muscularis propria into the subserosa, subsequently, providing an injury stimulus beyond the muscularis propria^5^. In our previous study, we identified six genes linked to fibrosis in the liver, kidney and IBD^18^. Two of these genes, *MGP* and *THBS2,* showed a particularly strong association with fibrostenosing CD. Matrix Gla protein (MGP) and CD36 (thrombospondin 2 (THBS2) receptor)^19,20^ are also markers that are differentially expressed in papillary and reticular fibroblasts of the skin^21^. Based on our previous evidence of MGP and THBS2 involvement in fibrosis in IBD, we decided to study them in different layers of the bowel wall to see if there is a similar stratification as observed in the skin. We hypothesized that subserosa in the bowel wall contains a fibroblastic subpopulation, which produces a strong fibrotic response to inflammatory stimuli that reach the subserosa and is distinct from the fibroblast populations of the lamina propria and submucosa. *MGP* and *THBS2* expression is regulated by several miRNAs, among them are* hsa-miR-135b*^22^*, hsa-miR-203a-3p*^23,24^, *hsa-miR-221-3p*^25–27^ and *hsa-miR-1246*^28,29^*,* which target* THBS2,* and *hsa-miR-143*^30^ and *hsa-miR-155-5p*^31–33^, which target *MGP.*

The aim of this study was to analyse fibroblasts in UC and CD, including immunohistochemical labelling of MGP and THBS2 protein as well as qPCR quantification of *THBS2* and *MGP* gene expression with their regulatory miRNAs in the submucosa and subserosa. Additionally, we used a digital pathology analysis to compare the collagen fibre characteristics of subserosal and submucosal fibrosis.

## Methods

### Patients

This exploratory observational retrospective study was performed on formalin-fixed-paraffin-embedded tissue samples taken from surgical resection specimens of the colon and ileum of 33 patients. There were 10 patients with CD (ileal and ileocecal resections), 12 patients with UC (colon resections) and 11 patients in the control group (resected for adenocarcinoma). Samples of CD were obtained from regions with most severe stenosis, samples of UC from regions with most active inflammation and samples for the control group were obtained from morphologically normal colon. The samples, primarily used for routine histopathological examination, were collected from the archives of the Institute of Pathology, Medical Faculty, University of Ljubljana.

After surgery, the resection specimens were transported fresh to the Institute of Pathology. At the Institute of Pathology, the resection specimens were opened longitudinally, rinsed and pined on polystyrene foam boards and fixed in 10% formaldehyde for 24 h. After fixation, the specimens were sampled for routine histological analysis. The major demographic data of the patient groups included in the study are summarized in Table 1.

**Table 1:** Major demographic data of patient groups. Kruskal–Wallis test for independent samples was used for statistics.

	Crohn`s disease n = 10	Ulcerative colitis n = 12	Control group n = 11
Sex (male:female)	7:3	7:5	7:4
Age (years)			
Age range	37–73	29–72	66–86
Median	37	29	66
*p* value vs. ulcerative colitis	0.46	/	0.001
*p* value vs. control	0.08	0.001	/
Disease duration (years)			/
Range	0–28	0–20	/
Median	5	3.5	/
*p* value vs ulcerative colitis	0.403	/	/
Indication for resection			
Fibrostenosis	10	0	0
Unresponsiveness to therapy	0	8	0
Fulminant colitis	0	4	0
Carcinoma	0	0	11

### Ethics

This study was approved by the National Medical Ethics Committee of the Republic of Slovenia (No. 0120-139/2019/4) and performed in accordance with the guidelines of the Declaration of Helsinki.

### Immunohistochemistry

For immunohistochemistry, we used antibodies against THBS2 (rabbit polyclonal, Abcam, UK, dilution 1/30), MGP (clone OTI11G6, Invitrogen, USA, dilution 1/1000), fibroblast activation protein (FAP) (clone EPR20021, Abcam, UK, dilution 1:100) and α-smooth muscle actin (α-SMA) (clone MA1-26017, Labvision, UK, dilution 1:20). Sections were treated with biotinylated secondary antibody and incubated with peroxidase conjugated streptavidin (iVIEW DAB Detection Kit, Ventana Medical System, Tucson, AZ, USA). Visualization was carried out with 3.3’ diaminobenzidine and counterstained with hematoxylin.

### Immunohistochemistry quantification

THBS2 and MGP expression in submucosal and subserosal fibroblasts was quantified by adapting the method described by Qiu et al.^34^. Intensity and percentage of positive fibroblasts were evaluated separately in the submucosa and subserosa of each tissue section. The percentage of positive fibroblasts was determined and assigned to one of five distribution categories: 0, < 5%; 1, 5–24%; 2, 25–49%; 3, 50–74%; 4, 75–100%. The intensity of staining was scored as 0 (negative), 1 (weak), 2 (moderate) or 3 (strong). The resulting distribution category and staining score were multiplied to produce a weighted intensity distribution score (0–12). THBS2 staining intensity was scored relative to smooth muscle as the internal reference (strong staining in the smooth muscle cells). MGP staining intensity was scored relative to endothelial cells as the internal reference (strong staining in the endothelial cells).

### Total RNA isolation

To compare the expression of fibrosis-related target mRNA and miRNA between submucosal and subserosal layers of the colon, the representative samples with well-characterised layers were selected. Using a 0.6 mm needle, 8–10 tissue samples of each layer were punched from tissue blocks. Total RNA was isolated using the MagMax FFPE DNA/RNA Ultra kit (A31881, Applied Biosystems, Thermo Fisher Scientific, Waltham, MA, USA) following the manufacturer protocol with the following amendment: protease digestion was performed overnight at 55°C with 15s shaking at 300 rpm every 4 min. The RNA quality was assessed using a NanoDrop 1000 spectrophotometer (Thermo Fisher Scientific) while the RNA concentration was determined using a Qubit Fluorimetric Quantification (Thermo Fisher Scientific) following their respective manufacturer protocols. Isolated total RNA was stored at -80°C.

### Quantitation of mRNA

#### Reverse transcription (RT) PCR

The OneTaq RT-PCR Kit (New England Biolabs, Ipswich, MA, USA) was used to synthesize cDNA following the manufacturer protocol. Briefly, a mixture of 3 µl total RNA (60 ng) and 1 µL Random Primer Mix were incubated at 70 °C for 5 min. Then 5.0 μL of M-MuLV Reaction Mix and 1.0 μL of M-MuLV RT-ase were added. The final 10 μL RT reaction volume was then incubated at 25 °C for 5 min, at 42 °C for 1h and at 80 °C for 4 min. cDNA was immediately preamplified and then stored at − 20 °C.

#### cDNA preamplification

Synthesized cDNA was preamplified using the TaqMan PreAmp master mix (4391128, Applied Biosystems; Thermo Fisher Scientific) following the manufacturer protocol. Briefly, a 10 μL preamplification reaction contained 2.5 μL cDNA, 5 μL 2× TaqMan PreAmp master mix and 2.5 μL 0.2× pooled TaqMan Gene Expression Assays (4331182 and 4351372, Thermo Fisher Scientific) (Suppl. Table 1). TE buffer solution (93302-500ML, Sigma Aldrich, Burlington, MA, USA) was used to dilute the pool of TaqMan GeneExpression Assays to 0.2 × concentration. Preamplification thermocycling parameters were: 95 °C for 10 min, 10 cycles of 95 °C for 15s and 60 °C for 4 min and; finally, 99 °C for 10 min. Preamplified cDNA was stored at − 20 °C.

#### Quantitative Real-Time PCR (qPCR)

The expressions of *IPO8, B2M, THBS2 and MGP* were analysed with qPCR whereby *IPO8* and *B2M* were used as reference genes (Suppl. Table 1). Each 10 μL qPCR reaction contained 4.5 μL of tenfold diluted preamplified cDNA, 5 μL of 2× Fast Start Essential DNA Probe Master (06924492001, Roche, Basel, Switzerland) and 0.5 μL of 20× TaqMan Gene Expression Assays (Suppl. Table 1). qPCR thermocycling parameters were: 50 °C for 2 min, 95 °C for 10 min and 40 cycles of 95 °C for 15 s and 60 °C for 1 min. Each qPCR reaction was performed in duplicates on Rotor-Gene Q System (Qiagen GmbH, Hilden, Germany).

qPCR reaction efficiency was determined using serial dilutions of pooled RNA samples for each group under the above-described conditions. Dilutions used were 5-, 10-, 25-, 125- and 625-fold. Each qPCR reaction to determine efficiency was performed in triplicates.

### Quantitation of miRNA

#### Reverse transcription (RT) PCR

miRCURY LNA RT Kit (Qiagen GmbH) was used to synthesize cDNA. A 10 μL RT reaction contained 2 μL total RNA (10 ng), 0.5 μL UniSp6 spike-ins, 1 μL 10× miRCURY RT Enzyme Mix, 2 μL 5× miRCURY RT Reaction Buffer and 4.5 μL RNase-free water. Each RT reaction was incubated at 42 °C for 60 min and at 95 °C for 5 min. If possible, cDNA was used immediately or it was aliquoted and stored at − 20 °C.

#### Quantitative real-time PCR (qPCR)

The expressions of *let-7e-5p, SNORD38B, miR-484, miR-221-3p, miR-1246, miR155-5p and miR-143-3p* were analysed with qPCR whereby *let-7e-5p, SNORD38B and miR-484* were used as reference miRNA (Suppl. Table 2). For qPCR of miRNA, miRCURY SYBR® Green PCR Kit (339347, Qiagen GmbH) was used according to manufacturer protocol. Briefly, each 10 μL qPCR reaction contained 3 μL of 60-fold diluted cDNA, 5 μL of 2× miRCURY SYBR^®^ Green Master Mix SYBR^®^ Green Master Mix, 1 μL 200× ROX Reference Dye and 1 μL 10× miRCURY LNA miRNA PCR Assay (339306, Qiagen GmbH) (Suppl Table 2). qPCR thermocycling parameters were: 95 °C for 2 min and 40 cycles of 95 °C for 10 s and 56 °C for 1 min. A melt curve analysis was performed in increments of 0.5 °C from 60 to 95 °C. Each qPCR reaction was performed in duplicates on QuantStudio 7 Pro (Thermo Fisher Scientific).

qPCR reaction efficiency was determined using serial dilutions of pooled RNA samples for each group under the above-described conditions. Dilutions used were 10-, 30-, 90-, 270- and 810-fold. Each qPCR reaction to determine efficiency was performed in triplicates.

### Statistical analysis

All data was analysed using the IBM SPSS Statistics for Windows, version 27.0 (IBM Corp., Armonk, NY). Relative gene expression was calculated using Cq averaged between duplicates of each sample. The averaged Cq values were then corrected to 100% qPCR efficiency as described by Kubista and Sindelka^35^. Using the method described by Latham et al.^36^, ΔCq was calculated by subtracting the Cq of the gene/miRNA of interest from the geometric mean Cq value of reference genes. Data distribution was tested for normality with the Shapiro–Wilk test. A paired t-test was used to determine significant differences in the expression of genes/miRNA between the submucosa and subserosa. Data homogeneity was tested using the Levene’s test. Brown-Forsythe and Welch’s ANOVA followed by Holm-Sidak or Dunnet’s T3 post-hoc analysis was used to determine significant differences between CD, UC and normal sample groups. All differences were defined as statistically significant if *p* < 0.05.

### Digital pathology

Histological sections were stained with Masson’s trichrome with aniline blue (04-01802, Bio-Optica, Milan, Italy) for detection of fibrosis. With this stain, cytoplasm stains red, nuclei black and collagen fibres blue. Digital Pathology images are acquired at 40 × (0.23 μm/pixel) using a Hamamatsu Nanozoomer Whole Slide Imager. FibroNest™ (PharmaNest, Princeton, NJ, USA), a cloud based high-resolution single-fibre image analysis platform, was used to quantify the fibrosis phenotype for (i) collagen content and structure features (12 traits), (ii) fibre morphometry (13 traits), and (iii) fibrosis architecture (7 traits to measure the organization of the fibres)^37,38^. Color normalisation and standardisation^39,40^ was performed to calibrate the images of the study. Each trait was quantified with 7 quantitative parameters (qFTs) to account for severity, distortion and variance, resulting in a total of 315 qFTs^41^. Fibres were also classified into Fine (smaller and less reticulated fibres) and Assembled (complex, larger and more reticulated fibre network). The qFT dataset was automatically surveyed to identify traits (principal qFTs) that would exhibit a meaningful (> 20%) relative difference (group average) between the submucosa and subserosa groups^42^. Such principal qFT are normalized to the tissue area and aggregated into a normalized “Crohn-Disease” Phenotypic Fibrosis Composite Score (CD-Ph-FCS) and displayed in the form of a heat chart (Fig. 4). This automated method has superior detection thresholds to detect biologically relevant differences in the histological phenotype of fibrosis due to severity^43–45^ or etiology^42^.

## Results

### α-smooth muscle actin immunohistochemistry

In normal colon, there was staining in pericryptal myofibroblasts in the lamina propria and smooth muscle cells in muscularis mucosae, muscularis propria and vascular walls.

**Figure 1:**
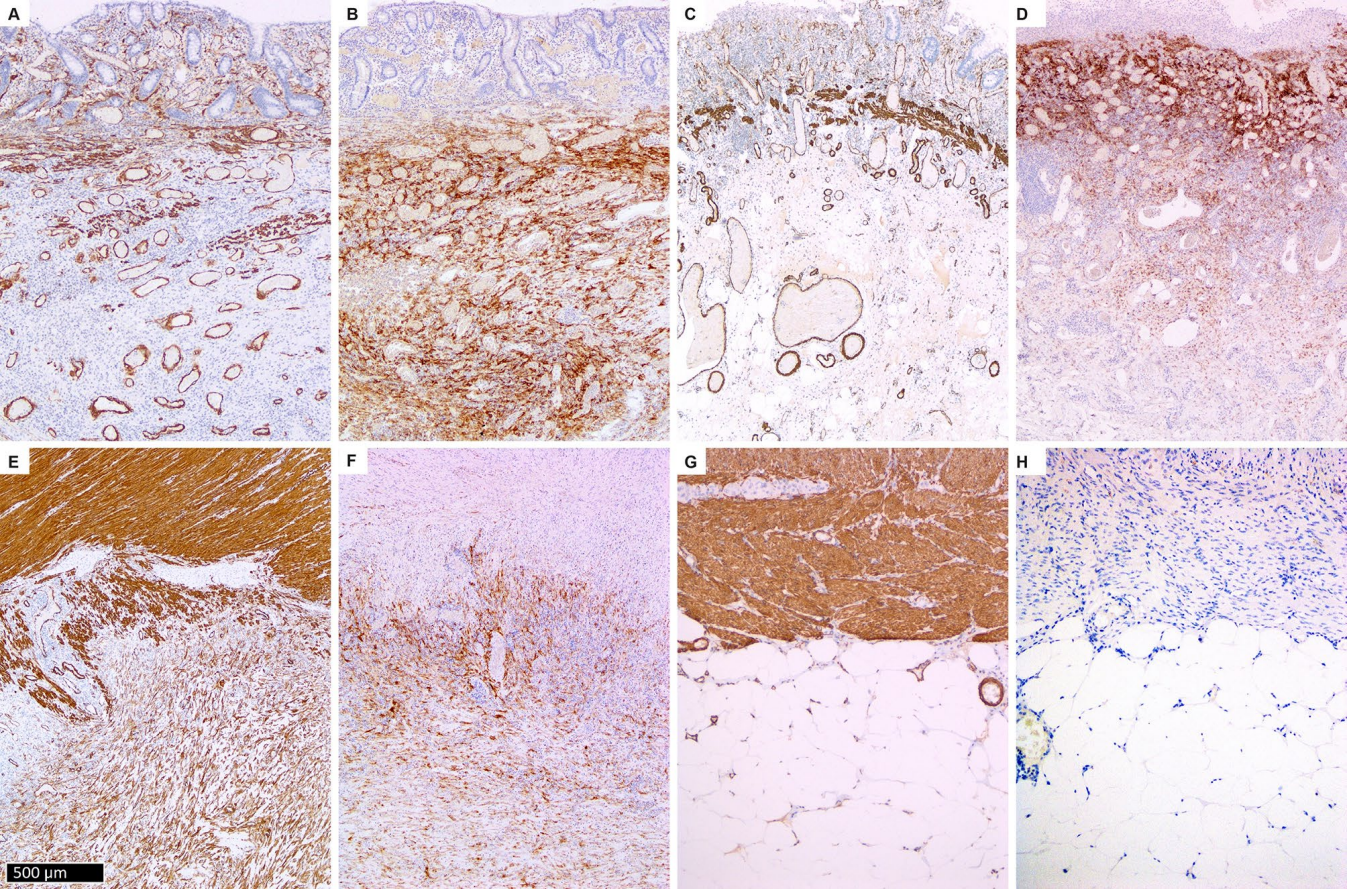
α-smooth muscle actin (α-SMA) and fibroblast activation protein (FAP) immunohistochemistry in Crohn’s disease and ulcerative colitis. Crohn’s diseases: α-SMA staining is present in normal structures (blood vessels and muscularis mucosae) in mucosa and submucosa (**A**). There is staining in muscularis propria and subserosal fibrosis (**E**). FAP staining is present in submucosa (**B**). There is staining in subserosal fibrosis but not in muscularis propria) (**F**). Ulcerative colitis. α-SMA staining is present in normal structures (blood vessels, muscularis mucosae, muscularis propria) (**C** and **G**). FAP staining is present in submucosa (**D**) but not in muscularis propria and subserosa (**H**).

In CD, there was staining in pericryptal cells in areas without ulceration, muscularis mucosae, muscularis propria and vascular walls. Additionally, there were relatively rare positive cells in the submucosa, lamina propria cells adjacent to ulcers and erosions and many positive spindle cells in areas of fibrosis in the subserosa (Fig. 1A and E).

In UC, there was staining in pericryptal cells in areas without ulceration, muscularis mucosae, muscularis propria and vascular walls. Additionally, there were relatively rare positive cells in the submucosa and lamina propria cells adjacent to ulcers and erosions. There was no staining in the subserosa outside vascular smooth muscles (Fig. 1C and G).

### Fibroblast activation protein immunohistochemistry

In the normal colon, there were no FAP+ cells.

In CD, there were many FAP+ spindle shaped cells in submucosal and subserosal fibrosis and few FAP+ spindle cells in muscularis propria. In the lamina propria, there were FAP+ cells in granulation tissue beneath erosions. In the submucosa, there were many more FAP+ cells than α-SMA+ cells, while, in the subserosa, the number of FAP+ and α-SMA+ cells was comparable, and they had the same spatial distribution (Fig. 1B and F).

In UC, there were many FAP+ spindle shaped cells in submucosal fibrosis and few FAP+ spindle cells in muscularis propria. In the subserosa, there were only rare FAP+ cells in the perivascular fibrous tissue. In the lamina propria, there were FAP+ cells in granulation tissue beneath erosions. There were many more FAP+ cells in the submucosa than α-SMA+ cells (Fig. 1D and H).

### Thrombospondin 2 immunohistochemistry

In the normal colon, THBS2 intensely stained the smooth muscle cells of muscularis propria and muscularis mucosae. There was also faint staining in cells corresponding to pericryptal myofibroblasts in the mucosa and rare spindle cells in the submucosa and in the outer layer of arteries.

**Figure 2:**
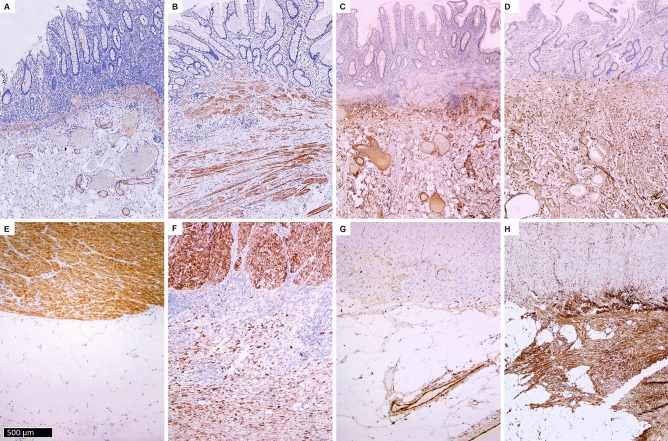
Thrombospondin 2 (THBS2) immunohistochemistry. No staining in submucosa in ulcerative colitis (**A**) and Crohn's disease (**B**) except in normal structures (blood vessels and muscularis mucosae). Staining of the muscularis propria and no staining in subserosa in ulcerative colitis (**E**). Staining of the muscularis propria and subserosal fibrosis in Crohn's disease (**F**). Matrix Gla protein (MGP) immunohistochemistry. Positive reaction in submucosa in ulcerative colitis (**C**) and Crohn's disease (**D**). No staining in subserosa in ulcerative colitis (**G**). S staining in subserosal fibrosis in Crohn's disease (H). There is staining in the blood vessel walls.

In UC, THBS2 showed a similar distribution to normal colon, with staining of smooth muscle cells and faint staining in pericryptal myofibroblasts and perivascular spindle cells. There were rare faintly stained spindle cells in areas rich with FAP+ cells in the submucosa. There were no or very few positive cells in the subserosa (Fig. 2A and E).

In CD, THBS2 showed a similar distribution as in UC. However, there were also numerous strongly positive cells that formed fibrosis in the subserosa, corresponding to myofibroblasts (FAP+, α-SMA+) (Fig. 2B and F).

The described patterns of staining were consistent across all studied samples (Supplementary Table 3).

### Matrix Gla protein immunohistochemistry

In the normal colon, MGP stained endothelial cells, the intima of arteries, as well as scattered spindle cells in the submucosa, subserosa, and perivascular spindle cells.

In UC, MGP stained endothelial cells, the intima of arteries, as well as scattered spindle cells in the submucosa, subserosa, and in perivascular spindle cells, as in normal colon. Additionally, there was an expansion of MGP+ spindle cells in the submucosa and a focal increase of MGP+ spindle cells around blood vessels in the subserosa (Fig. 2C and G).

In CD, MGP stained endothelial cells, the intima of arteries as well as scattered spindle cells in the submucosa, subserosa, and in perivascular spindled cells. Additionally, there was an increase in MGP+ spindle cells in the submucosa as well as positive staining in the myofibroblasts forming subserosal fibrosis (Fig. 2D and H).

The described patterns of staining were consistent across all studied samples (Supplementary Table 3).

### Expression of *THBS2* and *MGP* genes as well as their corresponding miRNAs in submucosa and subserosa in Crohn’s disease, ulcerative colitis and normal colon

**Figure 3:**
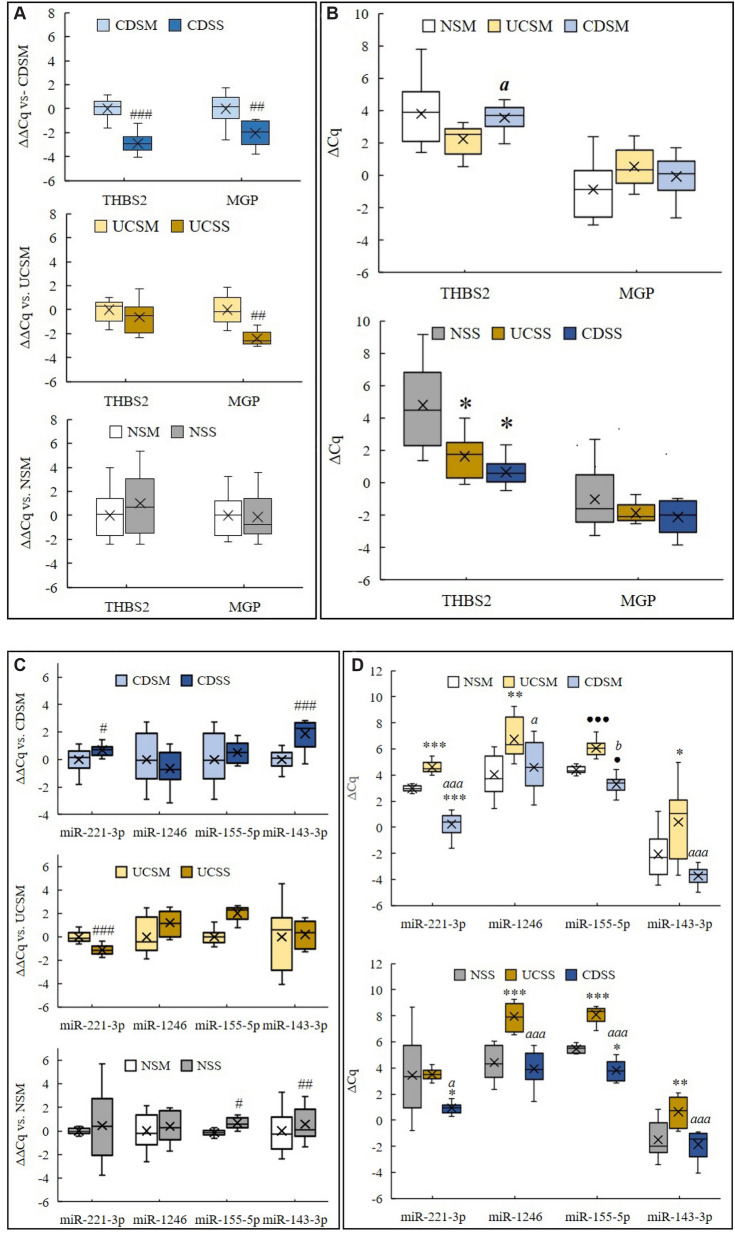
(**A**) and (**B**) *THBS2* and *MGP* gene expression determined by quantitative PCR. (**A**) *THBS2* and *MGP* gene expression in submucosa compared to subserosa in CD (n = 10), UC (n = 12) or normal colon (n = 11). Data presented as delta delta Cq normalised to the delta Cq of respective genes in submucosa. A paired T-test was used to determine significant differences at *p* < 0.01 (##), *p* < 0.001 (###). (**B**) *THBS2* and *MGP* expression profile. Data presented as the mean delta Cq ± standard deviation. One-way ANOVA with Dunnet’s T3 test were used to determine significant differences compared to the normal colon (n = 11) (*; *p* < 0.05) and between CD (n = 10) and UC (n = 12) (a; *p* < 0.05). (**C**) and (**D**) *THBS2* and *MGP* related miRNA expression determined by quantitative PCR. (C) *miR-221-3p*, *miR-1246*, *miR-155-5p*, *miR-143-3p* expression submucosal compared to subserosa in CD (n = 10), UC (n = 12) or normal colon (n = 11). Data presented as delta delta Cq normalized to the delta Cq of respective genes in submucosa. A paired T-test was used to determine significant differences at *p* < 0.05 (#), *p* < 0.01 (##), *p* < 0.001 (###). (D) *miR-221-3p*, *miR-1246*, *miR-155-5p*, *miR-143-3p* expression profile. Data presented as the mean delta Cq ± standard deviation. One-way ANOVA with Holm-Sidak test were used to determine significant differences compared to the normal colon (n = 11) (*; *p* < 0.05, **; *p* < 0.01, ***; *p* < 0.001) and between CD (n = 10) and UC (n = 12) (a; *p* < 0.05, aaa; *p* < 0.001) or with Dunnet’s T3 test to determine significant differences compared to the normal colon (n = 11) (●; *p* < 0.05, ●●●; *p* < 0.001) and between CD (n = 10) and UC (n = 12) (b; *p* < 0.05). *Legend* CD, Crohn’s disease; UC, ulcerative colitis; N, normal colon; SM, submucosa; SS, subserosa.

To determine the expression of *THBS2* and *MGP* genes, as well as their corresponding miRNAs, we performed a qPCR analysis. Both, *THBS2* and *MGP,* were significantly upregulated in the subserosa compared to the submucosa in CD, while only *MGP* was upregulated in the subserosa compared to the submucosa in UC (Fig. 3A). Interestingly, in the subserosa, only *THBS2* expression was significantly higher in CD and UC compared to the normal colon (Fig. 3B). Additionally, the expression of *THBS2* was significantly lower in the submucosa in CD than in the submucosa in UC (Fig. 3B).

Among *miRNAs* regulating *THBS2,* significant differences were observed in the expression of *hsa-miR-221-3p* and *hsa-miR-1246* (Fig. 3C and D), while the expression *hsa-miR-135b-5p* and *hsa-miR-203a-3p* was below detectable limits. Namely, the expression of *hsa-miR-221-3p* was significantly lower in the subserosa compared to the submucosa in CD, while it was significantly higher in the subserosa compared to the submucosa in UC (Fig. 3C). A significantly lower expression of *hsa-miR-221-3p* was observed in CD compared to UC and normal colon within both the submucosa and the subserosa, while a significantly higher expression was observed only in the submucosa in UC compared to the submucosa in normal colon (Fig. 3C). On the other hand, the expression of *hsa-miR-1246* was not significantly different when comparing the submucosa and the subserosa in CD, UC or normal colon (Fig. 3C). However, it was significantly lower in CD compared to UC and significantly higher in UC compared to normal colon within both the submucosa and the subserosa (Fig. 3D).

Among miRNAs regulating *MGP*, significant differences were observed in the expression of *hsa-miR-155-5p* and *hsa-miR-143-3p* (Figs. 3C and D). Namely, the expression of *hsa-miR-155-5p* was significantly lower in the subserosa compared to the submucosa in normal colon (Fig. 3C). Its expression was significantly lower in CD compared to UC and normal colon within both the submucosa and the subserosa (Fig. 3D). Additionally, the expression of *hsa-miR-155-5p* was significantly lower in UC compared to normal colon within both the submucosa and the subserosa (Fig. 3D). On the other hand, the expression of *hsa-miR-143-3p* was significantly lower in the subserosa compared to the submucosa in both CD and in normal colon (Fig. 3C). The expression of *hsa-miR-143-3p* was significantly higher in CD compared to UC and significantly lower in UC compared to normal colon within both the submucosa and the subserosa (Fig. 3D).

### Digital pathology analysis

**Figure 4:**
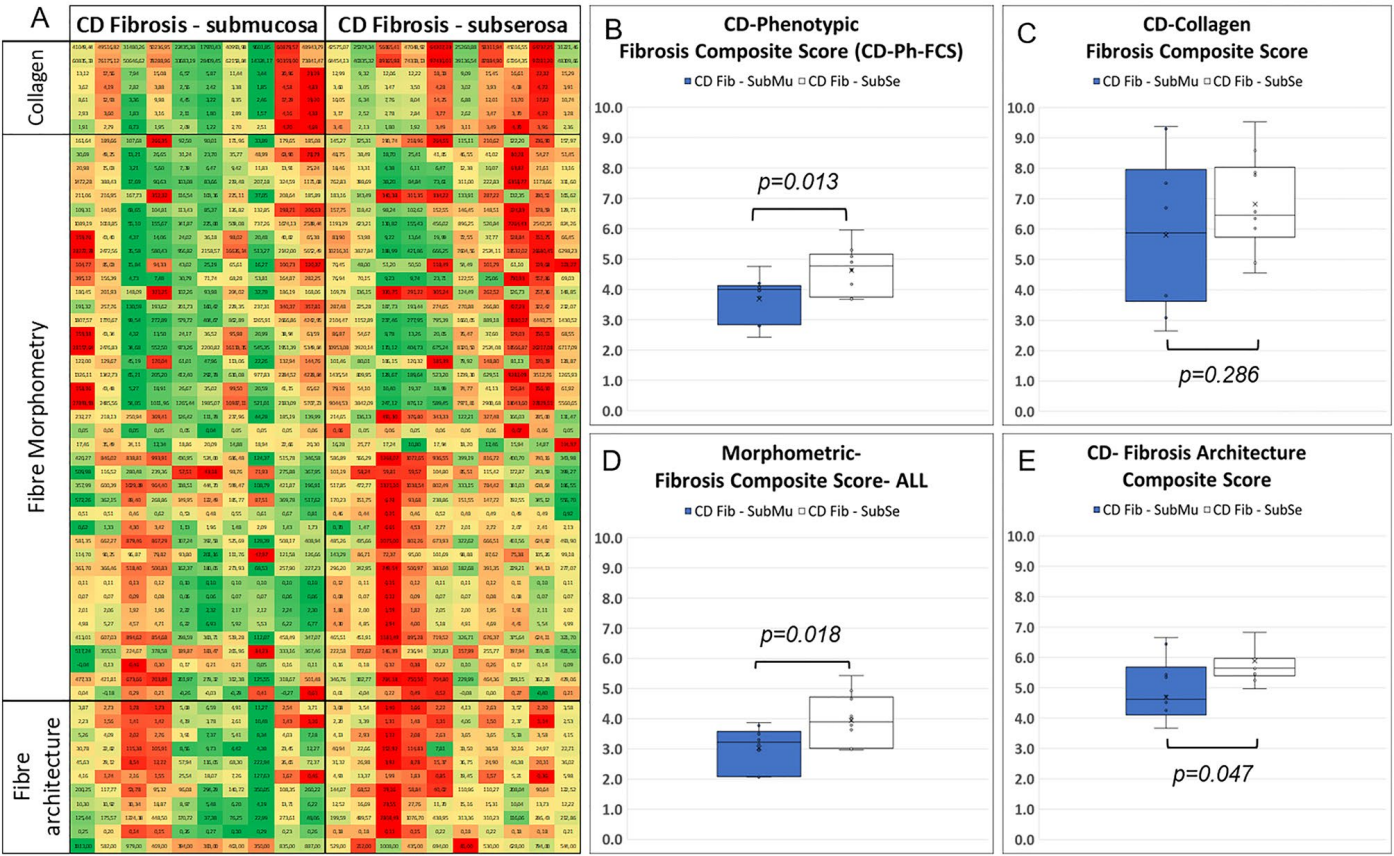
Fibrosis phenotypic differences in fibrosis in Crohn’s disease between the submucosa and subserosa layers. (**A**) Phenotypic chart. Every column represents a patient, and each a principal quantitative Fibrosis Trait selected by the artificial intelligence method to identify differences. (**B**) Principal qFTs are combined in a Composite Phenotypic Score CD-Ph-FCS. Panels (**C**,**D**,**E**) represent similar composite scores for the Collagen, Fibre Morphometric and Fibrosis Architecture subphenotypes (**A**–**E**: n = 10).

FibroNest Digital Pathology analysis assessed 139 principal quantitative fibrosis traits (principal qFTs) that describe the differences of the histological phenotype of fibrosis between the subserosa and the submucosa of CD fibrosis samples (Suppl Fig. 1). The related severity of each trait (green to red) is visually summarised in a phenotypic heatmap (Fig. 4A). The Phenotypic Fibrosis Composite Score formed from these principal qFTs showed statistically significant differences between the characteristics of subserosal and submucosal fibrosis (Fig. 4B). Similar composite scores can be calculated in each phenotypic layer (collagen content, fibre morphology and fibrosis architecture) (Fig. 4C–E), showing that the differences are explained by the morphometry of the fibres and their architecture, rather than the collagen content. The qFTs used to generate the composite scores describe fibre characteristics that include larger, thicker, more tortuous and reticulated fibres in the subserosa as compared to the submucosa. This novel quantitative approach is significantly superior to the conventional methods such as collagen content (Fig. 4E) that would not establish any difference.

## Discussion

We examined the expression of THBS2 and MGP in different layers of the bowel wall in IBD on protein, mRNA and miRNA levels with the hypothesis that the fibrotic response in the bowel wall shows distinct fibroblast populations across histologic layers as seen in skin fibrosis. Activated myofibroblasts were identified immunohistochemically with antibodies against α-SMA and FAP. The former is widely used as a marker of myofibroblasts^46^ but it also stains smooth muscles of the muscularis mucosae and muscularis propria, while the latter stains only myofibroblasts^47^. On the basis of morphology and immunophenotype, we were able to distinguish between smooth muscle cells (α-SMA+, FAP−) and active myofibroblasts (α-SMA+, FAP+) in the bowel wall.

Immunohistochemical analysis of THBS2 protein expression showed no or faint staining in the submucosa in both UC and CD as well as the subserosa in UC, but intense staining in subserosal fibrosis in CD. Immunohistochemical analysis of MGP showed positive staining in submucosal fibrosis in both CD and UC as well as subserosal fibrosis of CD, but no staining in the subserosa of UC.

qPCR analysis showed a significant upregulation of *THBS2* and *MGP* genes in the subserosa compared to the submucosa in CD. Additionally, *MGP* gene expression was significantly upregulated between the submucosa and the subserosa in UC, but not in the normal colon. The difference may be explained by focal areas of subserosal fibrosis in UC above the muscularis propria, which can be found in fulminant colitis^48^.

We also analysed the expression of regulatory miRNAs. We found downregulation of *hsa-miR-221-3p* in the subserosa compared to the submucosa in CD. *hsa-miR-221-3p* has been shown to inhibit *THBS2* expression^25–27^. In UC and normal colon where there is no subserosal fibrosis, *hsa-miR-221-3p* downregulation in the subserosa was not observed. In fact, it was upregulated in UC. Although *hsa-miR-1246* has also been shown to inhibit *THBS2*^28,29^, we did not observe significant differences between the subserosa and the submucosa in any of the groups, indicating other mechanisms might have a greater role in intestinal fibrosis. We also observed downregulation of *hsa-miR-143-3p*, which has been shown to inhibit *MGP* expression^30^, in CD and normal subserosa compared to the submucosa. Meanwhile, the expression of *hsa-miR-155-5p,* another miRNA that inhibits *MGP* expression^31,33^ showed significant differences between the subserosa and the submucosa only in normal bowel, but not in CD or UC. Unexpectedly, most tested miRNAs were downregulated in UC compared to both normal colon and CD in both the subserosa and the submucosa. The only exception was *hsa-miR-221-3p* expression, which was not significantly different between the subserosa in normal colon and UC. In addition to *THBS2* and *MGP,* these miRNAs have many other targets, which could potentially be of help in identifying pathways unique to UC.

Some cells in the submucosa of CD and UC were stained for α-SMA, which is a marker of smooth muscle and myofibroblasts^46^. However, much fewer cells were positive for α-SMA than FAP, which indicates that classical myofibroblasts represent a fraction of the expanded fibroblastic cell populations in submucosa (FAP+, α-SMA−). On the other hand, α-SMA+ cells were as abundant as FAP+ cells in the subserosa in CD, indicating that myofibroblasts represent most of the expanded fibroblastic cell populations in subserosal fibrosis (FAP+, α-SMA+).

Digital pathology analysis of the characteristics of subserosal and submucosal fibrosis in CD further confirmed that there is a significant difference in fibrosis in the two layers. The three subphenotypic scores show that the there are differences in the morphology and architecture of fibrosis in between layers, but not in collagen content. The subserosal fibrosis showed a higher proportion of more reticulated larger and thicker fibres in comparison to submucosal fibrosis, corresponding to a greater complexity of fibres and greater stiffness of the fibrosis in the subserosa^45^.

Myofibroblasts are widely accepted as the key cell type mediating fibrosis in various organs including the bowel wall in CD^6,46^; however, very little attention has been paid to the distribution of myofibroblasts in the fibrotic colon. Our study identifies activated myofibroblasts (FAP+, α-SMA+) in the subserosa indicating that injury of the subserosal compartment is necessary for the fibrostenotic reaction seen in CD.

In recent years, a microanatomically specific duality of fibroblast populations has been recognized in the skin with significant differences between papillary fibroblasts inhabiting the (superficial) papillary dermis and reticular fibroblasts inhabiting the (deeper) reticular dermis^49^. In skin during wound healing, reticular fibroblasts are activated first and, subsequently, mediate early extracellular matrix deposition and scar formation with loss of skin adnexa (and decreased functionality). On the other hand, skin papillary fibroblasts are activated later and mediate re-epithelization. Early activation or application of papillary fibroblasts promotes regeneration with less scar formation and regeneration of skin adnexa and functionality^9,11,50,51^. In wound healing and cell cultures, cells originating from reticular fibroblasts express α-SMA, while those originating from papillary fibroblasts do not^8,50^.

Our findings in colon show similarities to the stratification observed in the skin. We found a significant similarity in that there is a much greater expansion of fibroblasts in the subserosa than in the submucosa, with significant differences in the characteristics of the resulting fibrosis. qPCR experiments showed an upregulation of *THBS2* and *MGP* gene expression in the subserosa of CD fibrosis, as predicted by the differences observed in the skin. Additionally, we observed differential expression of α-SMA protein between subserosal (α-SMA+) and submucosal (mainly α-SMA−) fibroblasts, again mirroring the differences observed in skin. There is no consensus on the ideal marker to differentiate papillary and reticular fibroblasts of the skin, with many other potential markers, which could show a greater difference. Based on the differences we observed between subserosal and submucosal fibroblasts, we propose that the bowel could have a similar fibroblast duality with submucosal fibroblasts activated by superficial injury mediating regeneration with little scar formation and subserosal fibroblasts activated by injury of the deeper bowel wall mediating fibrosis/scar formation. Further studies are needed to confirm this hypothesis.

Much attention has been payed to different fibroblast populations of the skin and how they could be manipulated to achieve scarless healing after injury^52^. As a result, the fibroblast populations of the skin are much better studied than those of the intestinal wall. If we can confirm that the intestinal wall has similar fibroblast populations, with similar behaviour, we can transfer what we have learned from the skin to the process of intestinal fibrosis, helping us understand its pathogenesis and possibly transfer therapies developed for skin fibrosis to prevent fibrostenosis in the intestine. In contrast to skin, only a few studies have focused on different fibroblast populations in the intestinal wall. Kinchen et al.^13^ performed single cell sequencing on endoscopic biopsy samples and identified four subpopulations of fibroblast. However, by using endoscopic biopsies they were mostly limited to the fibroblasts of the lamina propria and possibly some of the submucosa without muscularis propria or subserosa. Recently, Mukherjee et al.^53^ performed single cell sequencing on resection specimens. They separated the samples in mucosa/submucosa tissue and muscularis propria tissue without mention of the subserosa. Mukherjee et al. identified several distinct fibroblast subtypes in the lamina propria and muscularis propria. They observed significant differences in the lamina propria subset between stenotic and non-stenotic CD and relatively small differences in muscularis propria subsets. Shu et al.^54^ performed single cell sequencing on mesenteric adipose tissue and creeping fat in stenotic ileal CD. They described a novel fibroblast population, which was annotated as inflammation associated fibroblasts. There is significant anatomical overlap between creeping fat and subserosal tissue; however, it is unclear if the study included tissue involved in the stenosis or adjacent fatty tissue. Additionally, there was no comparison to the other bowel layers. They described a profibrotic and proinflammatory tendency of creeping fat fibroblasts, which could overlap with the population we describe in the subserosa. The above mentioned studies do not have enough common points to confirm if that is the case. To the best of our knowledge, our study is the first to describe a difference in fibroblast populations based on submucosal and subserosal localization.

Our study has several limitations. It is purely observational, including a limited number of patients, studying only protein and gene expression, without functional confirmation. Additionally, fibroblast markers are not very specific, except FAP, as most markers react with several other cell types. This limitation has been somewhat reduced by careful correlation with morphology and different stains. However, a study which could employ additional advanced techniques such as flow cytometry with cell sorting and single cell sequencing would produce more reliable results.

## Conclusion

**Figure 5:**
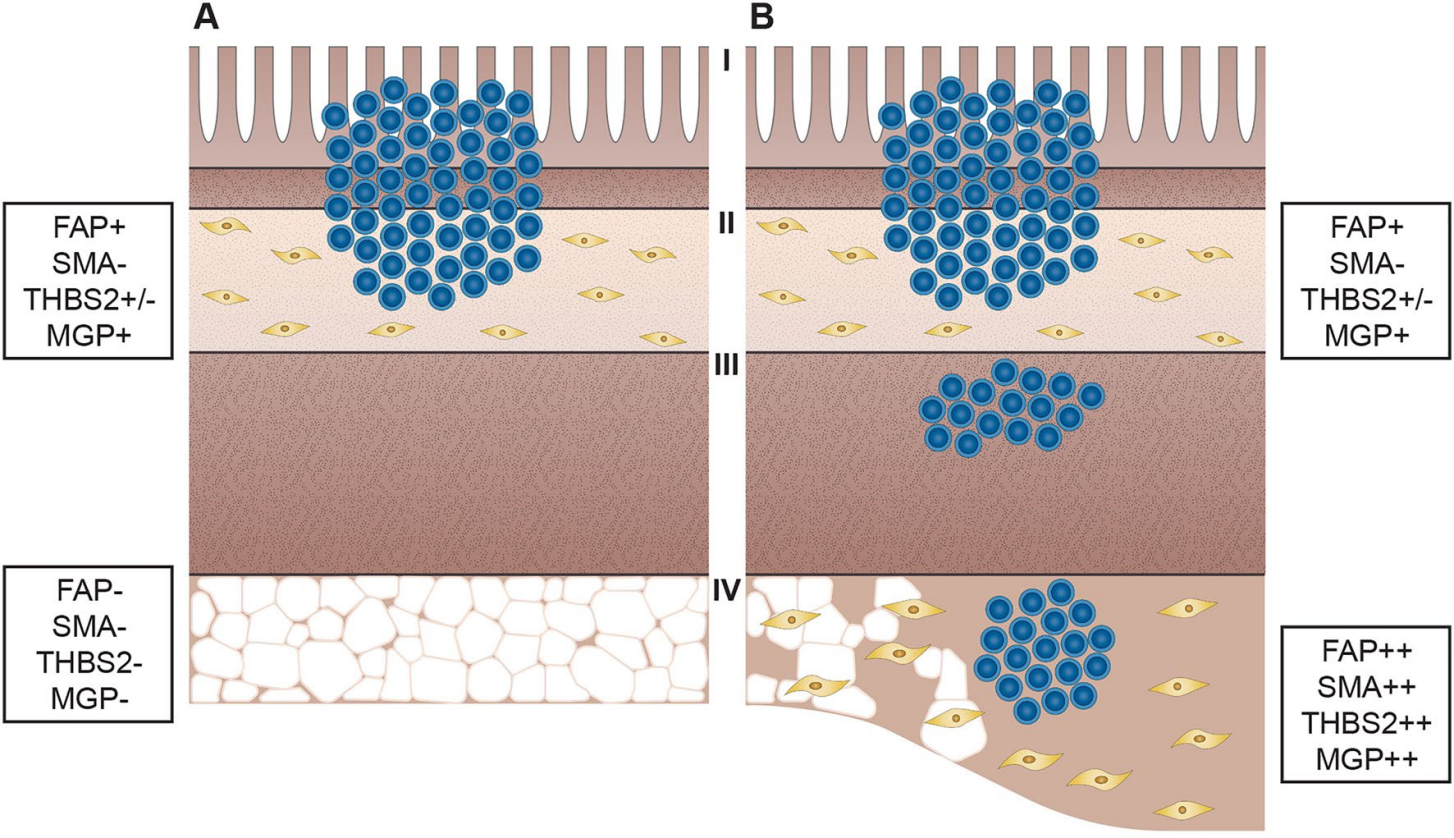
Schematic representation of inflammation and fibrosis in ulcerative colitis (UC) (**A**) and Crohn’s disease (CD) (**B**). Inflammation in mucosa and submucosa in UC and CD results in mild fibrosis with fibroblasts resembling those of the papillary dermis (FAP+, αSMA − , THBS2+/ − , MGP+*,* while inflammation in the subserosa in CD results in extensive fibrosis with fibroblasts resembling those of the reticular dermis (FAP++, αSMA++, THBS2++, MGP++). *Legend* FAP, fibroblast activation protein; αSMA, α-smooth muscle actin; THBS2, thrombospondin 2; MGP, matrix Gla protein; I, mucosa; II, submucosa; III, muscularis propria; IV, subserosa.

We observed differences in the submucosal and subserosal fibroblasts in fibrostenosing CD, suggesting distinct fibroblast populations in different layers of the bowel wall (Fig. 5). Submucosal and subserosal fibroblasts have similar markers as fibroblasts in skin in papillary and reticular dermis, respectively. Our findings provide pathogenetic explanation for the well-known fact that fibrostenosis develops in CD but not in UC: deep transmural inflammation in CD activating subserosal fibroblasts is needed to produce significant fibrosis. These results are further supported by image analysis of fibrosis in CD showing significant differences in the morphology and architecture of the collagen fibres in submucosal fibrosis in comparison to subserosal fibrosis. Our results contribute to the understanding of the pathogenesis of fibrosis in CD as well as in other diseases of the colon characterized by fibrostenosis, *e.g.,* the diverticulitis.

### Ethics approval statement

This study was approved by the National Medical Ethics Committee, Republic of Slovenia (No. 0120-139/2019/4) and carried out following the rules of the Declaration of Helsinki. Due to the study being done on archival tissue, the need for patient consent was waived. All authors have had access to the study data and reviewed and approved the final manuscript.

### Supplementary Information

Supplementary Information.

## Data Availability

The datasets used and analysed during the current study are available from the corresponding author on reasonable request.

## Abbreviations

α-SMA: α-Smooth muscle actin
CD: Crohn: s disease
FAP: Fibroblast activation protein
IBD: Inflammatory bowel diseases
MGP: Matrix Gla protein
qFT: Quantitative fibrosis trait
UC: Thrombospondin 2 (THBS2), ulcerative colitis
