# Supplementary material for: Thrombospondin 2, matrix Gla protein and digital analysis identified distinct fibroblast populations in fibrostenosing Crohn’s disease
Source: Sci Rep. 2024 Jun 14;14:13810. doi: 10.1038/s41598-024-64672-7 (PMC11178913; doi:10.1038/s41598-024-64672-7)
Supplement: Supplementary file 1 — Supplementary Information. [file 41598_2024_64672_MOESM1_ESM.docx]

**Supplementary Tables**

**Supplementary Table 1.** List of TaqMan Gene Expression assays

| TaqMan Gene Expression assays | Catalog number | Assay ID |
| --- | --- | --- |
| THBS2 | 4351372 | Hs01568054_g1 |
| MGP | 4331182 | Hs06636402_s1 |
| B2M | 4331182 | Hs99999907_m1 |
| IPO8 | 4331182 | Hs00183533_m1 |

**Supplementary Table 2.** List of miRCURY LNA miRNA PCR assays

| mirCury LNA miRNA PCR assays | Catalog number | Assay ID |
| --- | --- | --- |
| hsa-let-7e-5p | 339306 | YP00205711 |
| hsa-miR-484 | 339306 | YP00205636 |
| SNORD38B (hsa) | 339306 | YP00203901 |
| hsa-miR-221-3p | 339306 | YP00204532 |
| hsa-miR-1246 | 339306 | YP00205630 |
| hsa-miR-135b-5p | 339306 | YP00204130 |
| hsa-miR-203a-3p | 339306 | YP00205914 |
| hsa-miR-155-5p | 339306 | YP02119311 |
| hsa-miR-143-3p | 339306 | YP00205992 |
| UniSp6 | 339306 | YP00203954 |

**Supplementary Table 3.** Intensity distribution scores of immunohistochemical staining for THBS2 and MGP in fibroblasts in Crohn's disease, ulcerative colitis and control group.

*There are very few fibroblasts in the subserosa of the normal colon and ulcerative colitis; however, the majority of them strongly express MGP, which results in high intensiy distribution scores.

THBS2 = thrombospondin 2, MGP = matrix Gla protein

|  | **Crohn's disease** | **Ulcerative colitis** | **Control group** |
| --- | --- | --- | --- |
|  | n = 10 | n = 12 | n = 11 |
| **THBS2 submucosa** |  |  |  |
| Range | 0-4 | 0-4 | 0-2 |
| Median | 2 | 2 | 1 |
| **THBS2 subserosa** |  |  |  |
| Range | 6-12 | 0-4 | 0-2 |
| Median | 12 | 1 | 1 |
| **MGP submucosa** |  |  |  |
| Range | 9-12 | 9-12 | 9-12 |
| Median | 10.5 | 12 | 12 |
| **MGP subserosa** |  |  |  |
| Range | 9-12 | 9-12* | 9-12* |
| Median | 12 | 12* | 12* |
